# Success in restoring native plant communities on kimberlite mining dumps in the Afro‐alpine Drakensberg region of Lesotho

**DOI:** 10.1002/ece3.11022

**Published:** 2024-03-12

**Authors:** B. R. Ntloko, T. M. Mokotjomela, S. P. Mphafi, S. J. Siebert

**Affiliations:** ^1^ Letšeng Diamonds Cnr Kingsway and Old School Road Maseru Lesotho; ^2^ South African National Biodiversity Institute Free State National Botanical Garden Bloemfontein South Africa; ^3^ School of Life Sciences University of KwaZulu‐Natal Pietermaritzburg South Africa; ^4^ Unit for Environmental Sciences and Management North‐West University Potchefstroom South Africa

**Keywords:** grasslands, mining end‐land use, rehabilitation

## Abstract

Rehabilitation strategies for degraded mine dumps have generally seen limited success due to different complications associated with mining biophysical disturbance. In this study, we tested a combination of two methods to expedite revegetation of kimberlite tailings at Letseng Diamond Mine (i.e., in the Afro‐alpine areas of Lesotho). We ran trials on different growth media located on fine and coarse kimberlite tailings (i.e. sites) mixed with different substrate combinations and topsoil and sowing a seed mix comprised of native plant species. Overall, as predicted, fine kimberlite tailings displayed significantly higher plant abundance than coarse kimberlite tailings, and sown seeds performed significantly better than spontaneous colonisation by emerging species. Kimberlite tailings mixed with topsoil (100 mm) showed significantly greater plant abundance, and similarly, when coarse kimberlite tailings were introduced to fine tailings. Physicochemical analyses of growth media components suggested that topsoil provided additional nutrients and that plants could readily access available nutrients in the fine kimberlite tailings. We noted a gradual significant increase in plant abundance over 5 years, enhanced by new plant species emerging from the topsoil seed bank or by natural seed dispersal. Although plant abundance differed significantly, both fine and coarse kimberlite tailings displayed high plant species diversity (*H* = 3.4 and *D* = 0.95 and *H* = 3.5 and *D* = 0.95, respectively). Out of 36 emerging plant species, 15 species spontaneously colonised both growth media. The significant variation in abundance among plant species between treatments was mostly attributed to dominant forb species, namely *Chrysocoma ciliata*, *Glumicalyx montanus*, *Oxalis obliquifolia*, *Senecio inaequidens* and *Trifolium burchellianum*. We have identified suitable growth media for plant community restoration on kimberlite tailings in the Drakensberg alpine area using a seed mix of native plant species in combination with natural seed dispersal from the surrounding pristine environment. We provide evidence for using two complementary approaches to optimise native plant community development during restoration in the Drakensberg alpine area.

## Graphical abstract

Soil conditions are critical for achieving vegetation restoration. The use of plant seed mix enhances plant community development. Climax plant community development may take more than 5 years in the alpine grasslands.

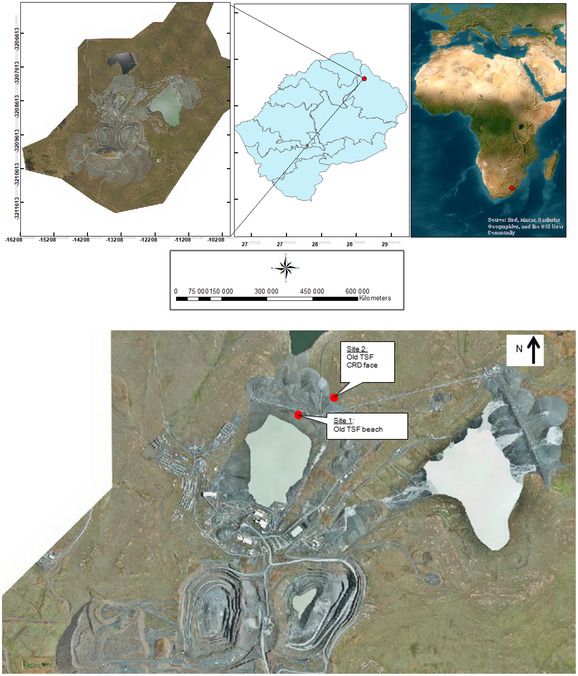

## 1 INTRODUCTION

Globally, mining is one of the important industries that boost economic development (Sun et al., 2022). However, mining activities lead to changes in land use and function due to excavations and infrastructural developments such as access roads, construction of buildings, equipment maintenance facilities and the associated waste materials (Espinosa‐Reyes et al., 2014; Haddaway et al., 2019; Khobragade, 2020). The known negative environmental impacts are numerous and include the disturbance of natural soil profiles, soil erosion, compaction, acidification, salinisation, solidification, reduced soil biota and changes in the local floral and faunal composition (Kuzevic et al., 2022; Mentis, 2020; Unanaonwi & Amonum, 2017). Other disruptions affect drainage patterns, which directly change the hydrological characteristics of the environment and ecosystem development processes (Khobragade, 2020).

Owing to the increasing environmental degradation caused by mining, land rehabilitation is essential to maintain the balance between environmental integrity and people's livelihoods (Broemme et al., 2015; Kabir et al., 2015; Rosa et al., 2016). Vegetation rehabilitation has been identified as a key solution for remediating disturbed landscapes and re‐establishing ecosystem goods and services for local people (Buisson et al., 2021; Carbutt & Kirkman, 2022; Hu et al., 2020; Mekuria et al., 2018). Plants stabilise degraded soil by binding soil particles through their root systems (Ranjan et al., 2015). Furthermore, the canopy cover created by vegetation aids in reducing and preventing the direct impacts of wind and water erosion (Sheoran et al., 2010; Zhang et al., 2014). As litter and debris from the re‐established vegetation decompose, it increases soil organic content, which in turn helps to enhance biological functionality and maintain different nutrient cycles (Zhang et al., 2014). High vegetation cover also improves many soil properties such as bulk density, aggregate stability and water and nutrient retention capacities, thus enhancing the overall fertility of the degraded areas (Mensah, 2015; Sheoran et al., 2010). In addition, rural socio‐economic development can be boosted through the development of nature‐based tourism, which is widely established on degraded farms that have been rehabilitated and turned into wildlife reserves, such as in the Eastern Cape Province, South Africa (Mokotjomela & Nombewu, 2020).

Generally, the cheaper and ecologically relevant way of stabilising mine tailings is through artificial revegetation using native plant species (Mendez & Maier, 2008; Piha et al., 1995) since it facilitates the reestablishment of natural vegetation (Festin et al., 2018; Mendez & Maier, 2008; Nyenda, 2020). Use of native seed material is a commonly preferred technique to support local biodiversity and the attainment of ecosystem services (Chamber of Mines of South Africa, 2007; Lu et al., 2017; Reid & Naeth, 2005; Toölgyesi et al., 2022). Studies have shown that the planting of selected pioneer species can be effective (Lu et al., 2017; Ntloko et al., 2022) because they often act as nurse species (Padilla & Pugnaire, 2006) and encourage the attainment of different ecological successional stages (Lu et al., 2017). It has been suggested that the optimal plant species for revegetation should be plant functional groups with similar responses to the environment and similar effects on ecosystem processes (Diaz & Cabido, 2001; Ostertag et al., 2015; Reid & Naeth, 2005). However, Rinella and James (2017) argued that plant establishment and persistence have often failed in this form of rehabilitation because of failure to select suitable plant species and limited attention to the preparation of media that will support plant performance (Reid & Naeth, 2005; Tripathi et al., 2016a, 2016b).

Successful rehabilitation of vegetation depends on several factors. Firstly, effective secondary natural vegetation recovery was enhanced where physical land destruction by machinery leaves behind soil that still contains some seed material, plant roots and spores for reproduction (Yunanto et al., 2019), and thus, highlights the importance of careful stockpiling topsoil containing plant propagules (Ntloko et al., 2022). Topsoil also provides nutrients, facilitates and improves water infiltration and retention on tailings and maintains adequate soil moisture content to support plant germination and growth and the establishment of biodiversity (Diop et al., 2022; Ntloko, 2020). Furthermore, soil resources maintain biogeochemical cycles and micro‐organisms which support plant establishment (Bhattacharyya, 2015; Lehman et al., 2015; Schoonover & Crim, 2015; Wang et al., 2017). Secondly, since natural recovery of the land and topsoil regeneration is slow, facilitation has been increasingly recognised as an important process in the recovery of plant communities in degraded lands (Brooker & Callaway, 2009; Zwiener et al., 2014). Thus, Ntloko et al. (2022) demonstrated that amelioration of kimberlite tailings with topsoil could encourage native plant establishment in the Drakensberg alpine area in Lesotho. However, the importance of proper soil handling techniques in mining areas is often overlooked during rehabilitation (Mhlongo & Amponsah‐Dacosta, 2015; van Rensburg & Maboeta, 2004). Thirdly, ambient environmental conditions and restoration targets influence rehabilitation success (Laughlin, 2014; Nyenda, 2020; Waldén et al., 2017), partly because site‐specific climate shapes the plant community assemblages (Chian et al., 2016; Reed et al., 2009).

Rehabilitation of kimberlite tailings storage facilities (TSF) is one of the obligations for effective mine closure at Letšeng Diamonds and the legislative environmental compliance needs and requirements of surrounding communities (see Ntloko, 2020). Two large priority surfaces targeted for rehabilitation at Letšeng Diamond Mine comprise the coarse and fine kimberlite tailings facilities. There is generally limited information and understanding of the establishment of vegetation in grasslands that have undergone severe disturbance (Vukeya et al., 2023). These shortcomings fail to achieve the desired rangeland use in the Lesotho Drakensberg.

In this study, we combined two strategies namely the use of native plant seed mixes and the amelioration of tailings with soil to determine the best media for vegetation rehabilitation of kimberlite tailing dumps in an alpine zone. This is the first study to determine which plant species are most suitable to colonise different mixtures of topsoil and mine waste materials of kimberlite rock. Our approach was aligned with restoration opportunities and targets that emphasised the reintroduction of native plant species and soil restoration of grasslands (Buisson et al., 2021).

We predicted that fine kimberlite tailings as the base would become better vegetated because of its close physical nature to alpine soil. Therefore, the objectives of the study were to determine:

1. if the application of native plant species seed mixes across different tailings and top soil treatments can promote revegetation of kimberlite tailings;
2. the contribution and abundance of seed bank emerging plant species in different tailings and top soil treatments; and
3. the role of physico‐chemical properties of tailings and soil mixes on plant establishment and abundance across the treatments.

## 2 MATERIALS AND METHODS

### 2.1 Study site: location, climate and vegetation

The study was conducted at Letšeng Diamond Mine (LDM) (29.0003° S; 28.8619° E) in the Mokhotlong District in the northeastern part of the Kingdom of Lesotho. Letšeng is situated in the priority conservation area of the Maloti‐Drakensberg Transfrontier Park and close to uKhahlamba Drakensberg World Heritage Site (Letšeng Diamonds, 2016). LDM is about 3400 m above sea level, making it the highest‐altitude diamond mine in the world (Lephatsoe et al., 2014). The mean daily temperature ranges from a minimum of 1.96°C to a maximum of 12.13°C as per LDM Weather Station. Snow is common during winter, especially from April to October when temperatures can drop as low as −20°C (Lephatsoe et al., 2014). The wind direction on the site is commonly westerly, with August and February being the months with the highest and the lowest wind speed, respectively. The mean wind speed is 5.97 ± 0.07 m/s at 10 m above ground level. The study area receives about 500–600 mm of rainfall per annum, most of which falls during the summer months, from October to March (Mucina & Rutherford, 2006).

**FIGURE 1:**
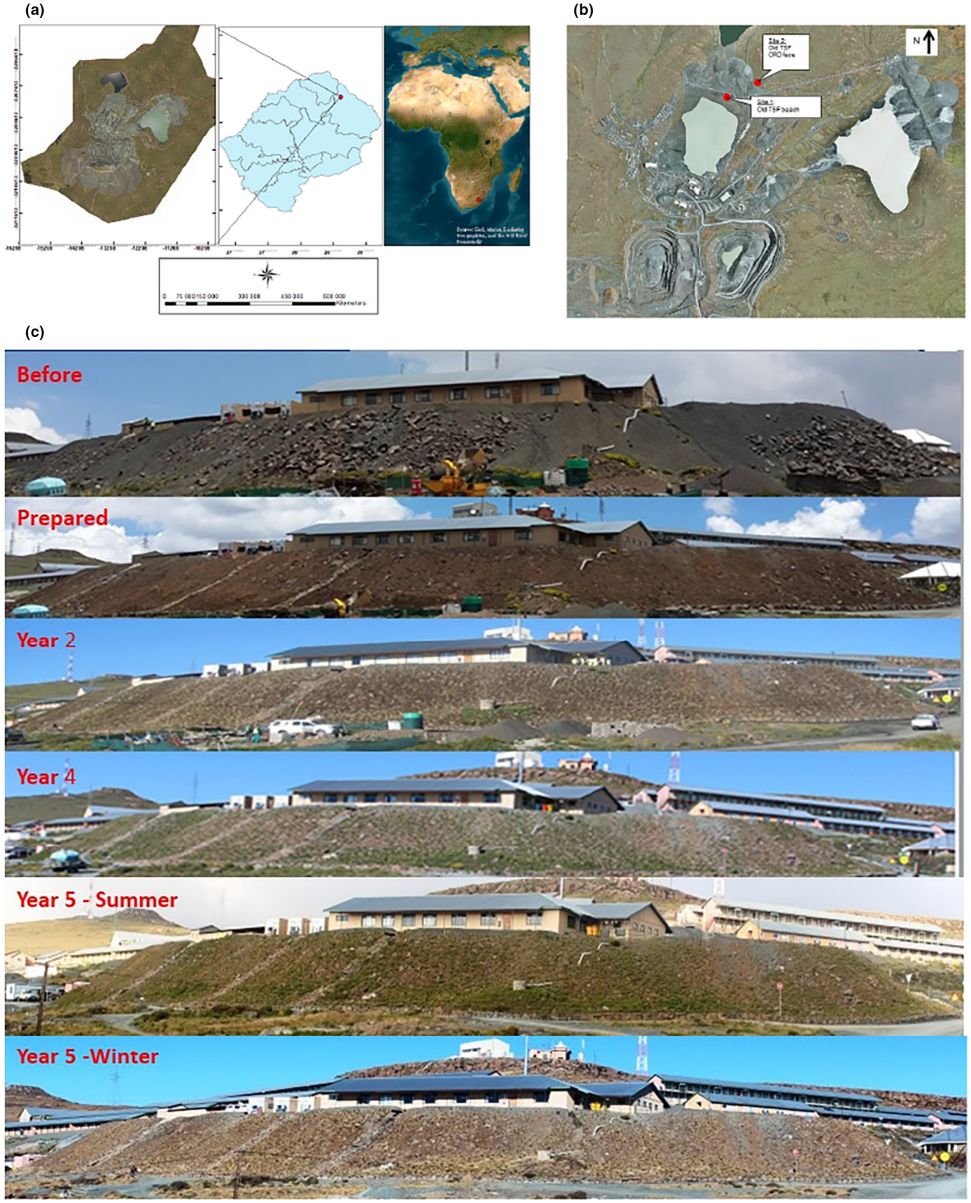
Location of Letšeng Diamond Mine in Lesotho, southern Africa (A), and the rehabilitation trial sites (B), Site 1 refers to the fine kimberlite tailings experimental site while Site 2 refers to the coarse kimberlite tailings experimental area. (C), Examples of the application of the study findings in rehabilitating the small slopes (Ntloko et al., 2022).

According to Mucina and Rutherford (2006), the study area falls within the Drakensberg Grassland Bioregion, specifically within the Drakensberg Afro‐alpine Heathland, with a portion consisting of Lesotho Highland Basalt Grassland. Vegetation change has occurred over decades, with past reports regarding the study area as a *Festuca* grassland (Staples & Hudson, 1938), with the dominant plant species being *Tenaxia disticha*, *Festuca caprina* and *F. scabra*; the latter is the most palatable for livestock grazing (Chakela, 1999). Currently, the fencing of the mining lease area has improved the vegetation cover compared with the adjacent areas, which are overgrazed (Letšeng Diamonds, 2016; Figure 1).

### 2.2 Geology

The geology of Lesotho consists of the Karoo Supergroup, which lies at the uppermost part of the Karoo basin (Schmitz & Rooyani, 1987). The Lesotho highlands are dominated by the Drakensberg Group (187–155 Ma), comprising basaltic lavas referred to as the Lesotho Formation, which forms the eastern highlands (Schmitz & Rooyani, 1987). The geology forms a notable range of highly elevated mountains known in Lesotho as the Maloti Mountains (the Drakensberg in South Africa). Dolerite dykes and kimberlite dykes cut through the sediments and lavas. Kimberlite is an intrusive igneous rock that can contain diamonds in its matrix and that is physically and chemically in sharp contrast to its host, basalt.

### 2.3 Soil material characteristics

#### 2.3.1 Pristine soil description

The United States Department of Agriculture (USDA) classification criterion was applied to the soils of the study site, a method which is preferred for its relevance in the Engineering Classification of Earth Materials, especially in contexts such as mining operations (USDA, 2012). Furthermore, by quantifying different parameters, the USDA criterion can allow decision‐making about plant life and other important aspects for guiding environmental rehabilitation (Baillie, 2001). Soil types in the pristine reference ecosystem at LDM are divided into four groups namely: shallow sandy loam soils, moderate to deep sandy loam soils, clayey soils with very little organic matter and soils that consist mainly of partially weathered rock fragments.

#### 2.3.2 Soil stockpile description

The topsoil used in this study was 3 years old—land stripping happened in 2012 and the experimental growth media were set up in 2014. According to the USDA criterion, the topsoil stockpile that was used in the growth media had a moderately coarse texture (sandy loam) with fine particles (i.e. silt and clay) accounting for 46.7%, whereas the proportion of coarse particles (i.e. sand) was 53.3%. The analysis of particle size distribution was done using the modified hydrometer method (Gee & Bauder, 1986; Mphafi, 2019).

#### 2.3.3 Physicochemical characteristics of the substrates

Representative samples of the fine and coarse kimberlite tailings, stockpiled soil and the reference site soil were collected from the designated areas. Samples of the stockpiled soil were collected using a soil auger at a depth of 30 cm from different topsoil stockpile sites around the mine and were then mixed thoroughly to make a composite sample. At the reference site, soil samples were also collected using a soil auger at 30 cm depth and at 10 m intervals of a Z‐shaped path across the width and length of the transect. Samples of the fine and coarse kimberlite tailings were collected from the TSF. Each sample was placed in clean airtight sample bags and transported to the Soil Science laboratory at the National University of Lesotho where they were stored in the refrigerator at 4°C to preserve their properties (Mphafi, 2019), whereas the other samples were air‐dried to 48 h. After drying, the mine waste residues were stored in the air‐tied containers without any further attempt to break them, while the stockpiled soil and reference site soil was crushed to pass through a 2 mm sieve prior to laboratory analysis.

Samples of all study materials were tested for selected physicochemical characteristics: pH, available nitrogen, organic carbon and electrical conductivity (EC; Mphafi, 2019). The analysis of pH was determined in distilled water and in KCl suspensions using the soil‐to‐solution ratio of 1:2.5 (following Kabała et al., 2016) and EC was determined by the saturation extract method (following Gharaibeh et al., 2021). Ammonium nitrogen, as an index of nitrogen (NH4+‐N) availability in the soil, was determined using the colorimetric method (Chapin et al., 2011), and available phosphorus was extracted with 0.5 M NaHCO_3_ (Wang et al., 2021) and analysed by molybdenate colorimetric method (Mphafi, 2019; Nielsen et al., 2017).

The exchangeable base cations (K^+^, Na^+^, Ca^2+^ and Mg^2+^) were leached with IN NH4OAc according to the procedure described by Thomas (1983) and determined with atomic absorption spectrometry (AAS) method (Knudsen et al., 1982; Mackay et al., 2005). The soil residues from the exchangeable base cations extraction were saturated with Na^+^ using an IN NaOAc solution whereby the excess Na^+^ was washed out with concentrated alcohol and the adsorbed Na^+^ displaced again with NH4OAc (Rhoades, 1993) and analysed by the AAS method (Knudsen et al., 1982) as an index of the Cation Exchange Capacity.

### 2.4 Growth media

#### 2.4.1 Fine kimberlite tailings

Fine kimberlite tailings are dominated by small particles (<10 mm) that originate as waste after processing for diamonds (Rankhododo, 2006). As a substrate it readily compacts, leading to a decrease in water and oxygen permeation in the soil (Lardner et al., 2011; Otieno & Ndoro, 2020). These conditions present a challenge for plant establishment (Lardner et al., 2011; Miller et al., 2021; Ntloko, 2020). Therefore, the first growth medium treatment comprised fine tailings as base material and experimental control.

A second treatment was composed of fine kimberlite tailings ripped in with 250 mm of coarse tailings to create a medium consisting of a rough texture as a way of reducing the reported compaction and the low permeability of the substrate to water and oxygen (Miller et al., 2021; Otieno & Ndoro, 2020). Coarse kimberlite tailings have relatively larger particles (10–55 mm) and originate from the diamond processing plant. It was anticipated that this treatment would allow plant roots to penetrate the substrate (Reid & Naeth, 2005; Rouble, 2011).

The third treatment was created by ripping coarse kimberlite tailings into the fine kimberlite and further mixing it with topsoil to create a functional growth medium that could create a habitat for plants (Drozdowski et al., 2012; Lardner et al., 2011). It has been shown that ripping reduces soil compaction and increases root performance in plant growth (Lardner et al., 2011). The topsoil in the study site is renowned for its very low organic matter content (Ntloko, 2020).

The fourth treatment was similar to the third treatment, but we increased the volume of coarse kimberlite tailings to stabilise the slope against water erosion (Reid & Naeth, 2005; Rouble, 2011) and to determine whether there were any interactive effects between the course and fine tailings. The addition of coarse‐grain material in compacted soil such as the fine kimberlite tailings in this study can increase plant performance through improved soil structure (Rouble, 2011).

#### 2.4.2 Coarse kimberlite tailings

The first treatment comprised the coarse kimberlite tailings as base material and was regarded as an experimental control treatment. Coarse kimberlite tailings are characterised by high drainage ability owing to their high porosity (Copeland & Teixeira, 2019; Dhar et al., 2022), and together with a limited medium for root formation, create a hostile environment for vegetation establishment (Ntloko, 2020).

Soils that have high infiltration rates cannot retain dissolved nutrients and thus have high drainage potential and poor soil fertility levels (Naeth & Wilkinson, 2010; van Rensburg & Maboeta, 2004). Therefore, the second treatment used coarse kimberlite tailings ameliorated with topsoil to improve the substrate structure, water retention, and nutrient availability for plants to become established (Dhar et al., 2022; Miller, 2019; Stanton‐Kennedy, 2008).

Previous studies have shown that kimberlite tailings on their own are unstable and highly erodible, thus posing a risk to the establishment of plants and potentially causing them to fail (Alborov et al., 2020). Therefore, the third treatment was created with coarse kimberlite tailings mixed with waste rock. Such a combination increases surface structure and attenuate soil erosion while creating a habitat for colonisation and the establishment of plant communities (Rouble, 2011).

The last treatment was made up of coarse tailings, mixed with waste rock with additional topsoil, to create a hybrid medium consisting of the combined functionality from the other treatments.

### 2.5 Seeding trials

A representative mass of 100 seeds replicated five‐fold from each of nine different plant species was used to create a proportional seed mix of equal contributions per species (see West et al., 2012). The nine plant species chosen included *Tenaxia disticha* which was shown to stabilise kimberlite tailings (Ntloko et al., 2022), whereas other subsidiary plant species (Table 1) were selected based on the pre‐adaptation (i.e. morphological traits, Graff & McIntyre, 2014) to the local environmental conditions, including those of the mine site (Ntloko, 2020).

**TABLE 1:** List of native plant species used in the seed mix.

Species name	Family	Life/growth form
*1. Sisymbrium turczaninowii* Sond.	*Brassicaceae*	Annual, therophyte
*2. Athrixia fontana* MacOwan	*Asteraceae*	Perennial, cushion
*3. Cotula paludosa* Hilliard	*Asteraceae*	Perennial, cushion
*4. Dierama robustum* N.E.Br.	*Iridaceae*	Perennial, geophyte
*5. Helichrysum trilineatum* DC.	*Asteraceae*	Perennial, cushion
*6. Hesperantha schelpeana* Hilliard & B.L.Burtt	*Iridaceae*	Perennial, geophyte
*7. Kniphofia caulescens* Baker	*Asphodelaceae*	Perennial, geophyte
*8. Selago flanaganii* Rolfe	*Scrophulariaceae*	Perennial, cushion
*9. Tenaxia disticha* (Nees) N.P.Barker & H.P.Linder	*Poaceae*	Perennial, tussock

Equal amounts of seed mix were hand‐casted in the experimental plots located in the fine and coarse kimberlite tailings and subsequently manually raked into the soil in October 2014 (see Ntloko, 2020; Ntloko et al., 2022). Our seeding rate was partly derived from the recommended seeding rate of 7 g/10 m^2^ (7 kg/ha) as determined for plant species (E‐Tek Consulting, 2013), taking into consideration that the viability of naturally collected seeds is highly variable (Rodríguez‐Arévalo et al., 2017). In addition, this seeding rate was based on complex site‐specific conditions characterised by high physical disturbance from mining operations and aimed to effectively offset the possibility of high seedling mortality. Above all, high seeding rates of ground cover species such as grasses are recommended during mine rehabilitation to protect the tailings from eroding (Maliba et al., 2011).

There was no irrigation for the duration of the experiment. Seedling germination data were collected weekly, starting on the first date of seedling emergence in each treatment until the time when no new seedlings were observed (Bathusi Environmental Consulting cc, 2020; Ntloko et al., 2022). Unknown seedlings were identified using imagery reference features obtained from the pristine vegetation patches in the study site and from the Pooley (2003) floral field guide. Since the use of seeding during restoration often results in failed establishment and persistence (Rinella & James, 2017), the seeds sown in the experimental plots were allowed to naturally break dormancy, germinate, and develop into young plants over 2 years before commencing with the comparative study (i.e. 2014–2015; Ntloko et al., 2022).

From February 2016 to December 2020, we monitored plant recruitment and colonisation for plant community development by counting the number of plants per species per plot per treatment as a surrogate measure of plant abundance and determined the overall species diversity by monitoring each plot over 5 years (i.e. 2016–2020). The average size of each plot was 300 ± 17.8 m^2^.

In each month of the year, when possible, plots were sampled in a systematic approach. Concurrently, we recorded the emergence of additional non‐sown plant species in our original baseline plant community, which was made up of nine plant species in the seed mix. Records of species were determined up to species and family levels, and species were categorised as original species (OR) if they were manually sown in the trial plots, and as emergent species if they arrived in the natural course of events, such as either from the soil seed bank or from seed dispersal.

### 2.6 Statistical analysis

To determine the plant abundance per growth medium and to compare the growth media, the plant count data were analysed using a generalised linear model (GLM), fitted with Poisson error distribution, as a typical counts data statistical model, using SPSS version 28 (IBM Corp., Armonk, NY, USA). The counts of the number of plants in each plot per site were specified as the quantitative response variable for all sites. The growth medium (i.e. the treatment) and the years of observation (i.e. whether sown or from the soil seed bank) were specified as the predictor variables.

To determine the extent of plant community development, we calculated the total plant species diversity using the Shannon‐Wiener and the Simpson diversity indices for each site. To confirm that colonisation had occurred, our calculations only focused on emergent plant species recorded in each treatment (i.e. the growth medium), whereas the other nine species were used as a baseline for facilitating plant colonisation (Corbin & Hall, 2012; Vaughn et al., 2010).

To determine differences between the sampled material, all the measured physicochemical properties of the soils and kimberlite tailings were subjected to General Linear Model Analysis of Variance (GLM‐ANOVA) using SPSS version 28. Each sampled material was referred to as a treatment with a distinct name and specified as the predictor variable while all measured parameters were specified as response variables. The significant differences between the means were determined using the Duncan multiple range test at the probability level of at least 0.05 (*p* ≤ .05).

## 3 RESULTS

### 3.1 Plant abundance in fine kimberlite tailings versus coarse kimberlite tailings

**FIGURE 2:**
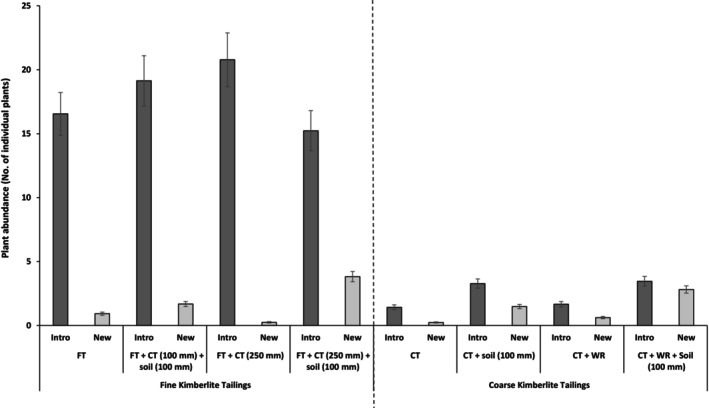
Total plant abundance per plot in different growth media treatments comprising fine and coarse kimberlite tailings. CT, coarse kimberlite tailings; FT, fine kimberlite tailing; WR, waste rock; and ‘soil’ refers to topsoil from stockpile. ‘Intro’ = seedlings from introduced original seed mix and ‘New’ = seedlings from self‐colonisation. Bars represent the ± standard error of the mean.

Overall, the fine tailings treatments displayed significantly higher plant abundance than the coarse tailings (Wald χ^2^ = 831.9; df = 1; *p* < .001). Different growth media in each site (fine vs. course tailings) displayed a significant difference in respect of plant abundance (fine tailings: Wald χ^2^ = 62.6, df = 3, *p* < .001 and course tailings: Wald χ^2^ = 199.8, df = 3, *p* < .001), with the growth media that had topsoil (100 mm) added showing significantly greater plant abundance (Figure 2, see Table S1). Noteworthy was that there were significant interactions between the sites and category of plant species, whereby introduced original plant species (i.e. sown) were more abundant than the emerging species (i.e. seed bank) in both fine and coarse kimberlite tailings growth media (Wald χ^2^ = 16.8, df = 7, *p* = .019; Figure 2) even though the fine tailings still had greater abundance.

**FIGURE 3:**
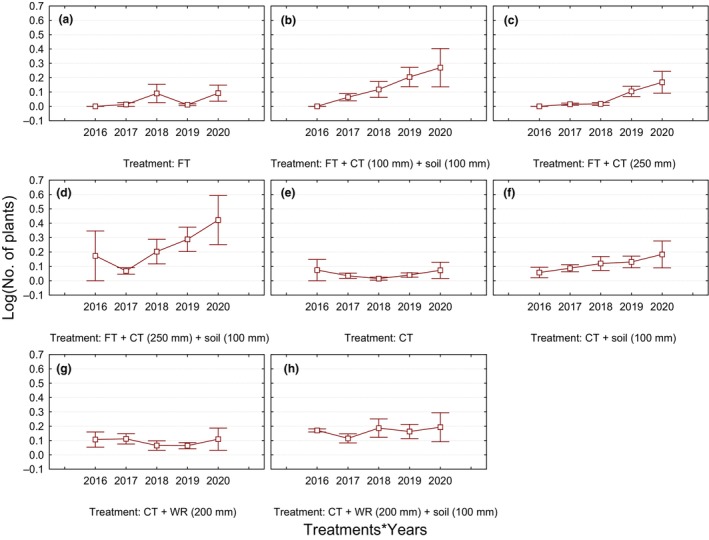
Variation in plant abundance in different treatments (growth media) comprising fine and coarse tailings from 2016 to 2020 for emergent plant species originating from the soil seed bank or natural dispersal. CT, coarse kimberlite tailings; FT, fine kimberlite tailings; ‘soil’ refers to topsoil; WR, waste rock. Bars represent the ± standard error of the mean.

Overall, the fine kimberlite tailings also showed significantly greater plant abundance for emergent plant species than the coarse kimberlite tailings (Wald χ^2^ = 6.1, df = 1, *p* = .011). There were also significant differences among years, showing a gradual increase in the abundance of plants contributed by emergent plant species from the topsoil seed bank and natural seed dispersal from surrounding natural grassland (fine tailings: Wald χ^2^ = 105.5, df = 4, *p* < .001 and coarse tailings: Wald χ^2^ = 217.7, df = 4, *p* < .001; see Table S1), with the year 2020 showing the highest plant abundance (Figure 3b,d). Coarse kimberlite tailings had a significant and positive effect on fine kimberlite tailings with respect to plant abundance (Figure 3c). The patterns were similar between both sites, although the fine tailings sites had significantly higher plant abundance; and it is worth noting that, as was expected, the experimental controls (comprising fine and coarse kimberlite tailings‐only treatments) poorly supported plant life across the years (Figure 3a,e; see Table S1).

### 3.2 Species diversity indices

Overall, both the fine and the coarse kimberlite tailings displayed relatively high plant species diversity, as suggested by the two diversity measure scores (Table S2). Fine and coarse tailings had a similar Shannon‐Wiener index (*H* = 3.4 and 3.5. respectively) and an identical Simpson's Index (*D* = 0.95) for all treatments combined.

**FIGURE 4:**
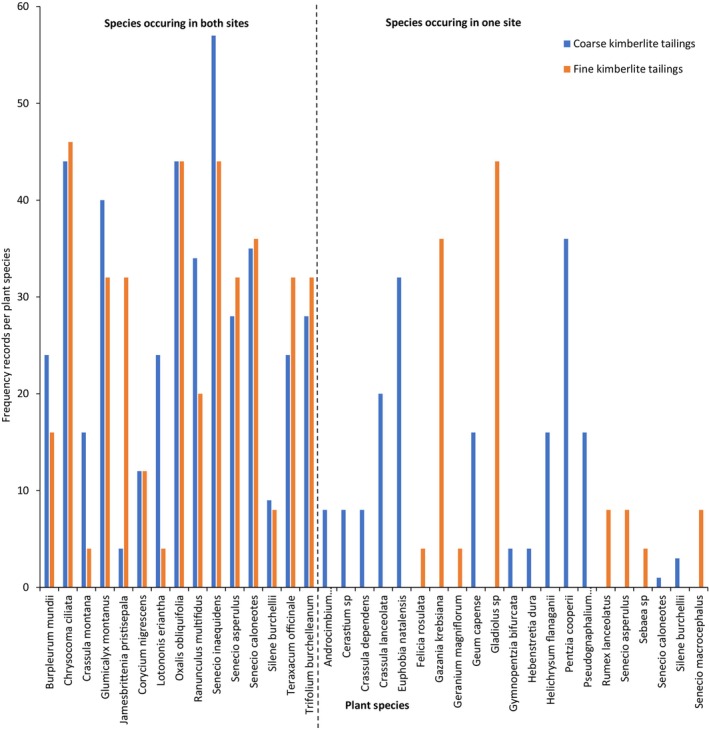
Abundance of plant species spontaneously colonising treatments of fine and coarse kimberlite tailings. Plant species frequency ranged from 0 to 56 records. The dotted line separates the species that colonised both sites and those that were recorded in either one of the sites.

Out of a total of 36 emergent plant species, 15 species were recorded in both growth media (Figure 4). Overall, different plant species displayed significantly different abundance with respect to their frequency records in both the fine and the coarse kimberlite tailings (Pearson χ^2^ = 404.2; df = 50; *p* < .001; Figure 4). The dominant plant species in both growth media were *Chrysocoma ciliata* L., *Glumicalyx montanus* Hiern, *Oxalis obliquifolia* Steud. ex A.Rich., *Senecio inaequidens* DC., and *Trifolium burchellianum* Ser. (Figure 4).

### 3.3 Physicochemical characteristics of the substrates

Overall, most physicochemical attributes of the fine and coarse kimberlite tailings were not significantly different except for the available phosphorus which was more in the coarse tailings (*F*_(1,56)_ = 15.3; *p* = .017) but had significantly less magnesium than the fine tailings (*F*_(1,56)_ = 10.0; *p* = .032; Table 2). As expected, the stockpiled soil used in this study had significantly lower organic carbon than natural soil obtained from non‐disturbed areas, but it remained significantly greater than the fine and coarse tailings (Table 2).

**TABLE 2:** The physicochemical attributes of the fine and coarse kimberlite tailings and the soils ± standard error of the mean of the samples.

Properties	Fine kimberlite tailings	Coarse kimberlite tailings	Stockpiled soil	Reference soil (pristine)
pH_water_	7.64 ± 0.17^a^	7.75 ± 0.28^a^	6.99 ± 0.01^a^	6.83 ± 0.06^a^
pH_KCl_	7.39 ± 0.09^a^	7.34 ± 0.04^a^	6.65 ± 0.02^a^	6.72 ± 0.09^a^
EC (dS/m)	0.23 ± 0.01^a^	0.42 ± 0.37^a^	0.36 ± 0.01^a^	0.23 ± 0.03^a^
${{\mathrm{NH}}_{4}}^{+}$ (mg/kg)	0.25 ± 0.02^a^	0.16 ± 0.06^a^	0.25 ± 0.02^a^	0.83 ± 0.06^b^
P^3−^ (mg/kg)	0.41 ± 0.09^a^	2.72 ± 0.18^b^	20.70 ± 1.73^c^	25.90 ± 2.25^c^
OC	1.4 ± 0.4^a^	1.2 ± 0.4^a^	1.83 ± 0.14^a^	3.14 ± 0.06^b^
Base cations (cmol/kg)				
Ca^2+^	15.52 ± 2.02^a^	12.69 ± 1.81^a^	10.74 ± 1.23^a^	9.18 ± 0.58^a^
Mg^2+^	1.75 ± 0.17^a^	0.84 ± 0.08^b^	2.91 ± 0.13^c^	2.23 ± 0.06^c^

*Note*: Letters denote significant differences between the samples' means.

Abbreviations: EC, electric conductivity; OC, organic carbon.

## 4 DISCUSSION

The rehabilitation of mine waste dumps is a legal obligation globally (Kabir et al., 2015; Nyenda, 2020; Rosa et al., 2016). Since many vegetation rehabilitation projects often suffer from poor plant persistence in mine dumps (Rinella & James, 2017), we combined two approaches. We ameliorated the kimberlite tailings with local topsoil and used a seed mix comprising nine tested native plant species as a way of facilitating plant community development. The complementarity of these two approaches was evident and was effective in achieving a stable ecological plant community on the kimberlite tailings of an Afro‐alpine area.

Fine kimberlite tailings displayed significantly higher plant abundance than the coarse kimberlite tailings, with or without topsoil mixes, and may be attributed to the differences in the availability of important nutrients for plants between the two sites (Jones et al., 2023; Reid & Naeth, 2005). While an absence of organic carbon and a lack of available macronutrients are principal limitations to plant colonisation (Reid & Naeth, 2005), we found that fine kimberlite tailings had relatively more available phosphorus which is important for seed germination, root development and generation of proteins which carry energy to the plant cells and are needed for photosynthesis (Khan et al., 2015). Also, fine kimberlite tailings have small particles that might display characteristics and functions very similar to that of soil (Swami et al., 2007), such as retention of soil moisture (Dhar et al., 2022; Reid & Naeth, 2005). For successful revegetation, kimberlite tailings require structural and chemical amelioration to make them habitable by plants during rehabilitation (Drozdowski et al., 2011; Reid & Naeth, 2005). Thus, higher plant abundance and colonisation were observed for fine kimberlite tailings ameliorated with topsoil (van Rensburg & Maboeta, 2004). Topsoil mixes provide additional soil nutrients and organic matter, which is known to be limiting in kimberlite tailings (Reid & Naeth, 2005). Soil also improves the temperature for microbial activities that are essential for soil health, and thus plant health (Dhar et al., 2022; Miles & Tainton, 1979; van Rensburg & Maboeta, 2004).

We can therefore ascribe the better performance on fine tailings to its structure, which enhances the root environment, and its superior nutrient status. Substantial carbonation is reported for coarse kimberlite tailings (Jones et al., 2023), and the milling thereof during the extraction process to form fine tailings, may have enhanced the fertility of the latter. Organic carbon is important in soil fertility and has multiple roles such as binding of soil particles, development of soil structure, adsorption of nutrients and water retention (Kalshetty et al., 2014; Musinguzi et al., 2013). Fine tailings also have more magnesium and calcium than coarse tailings and they are considered important components of the carbonation process reported by Jones et al. (2023), while also preferred for plant physiological wellbeing (Khan et al., 2015). Ripping the two substrates into each other created functional microsites for plant colonisation (Drozdowski et al., 2012; Lardner et al., 2011; Riis, 2008) because ripping reduces soil compaction and increases gaseous exchange and plant root performance (Lardner et al., 2011; Reid & Naeth, 2005). Since all growth media with top soil additions had high plant abundance and colonisation, we contend that emergence of new plant species might also be partly explained by soil seed banks and natural seed dispersal, as reported for Australian mines (Mifsud et al., 2010). The continuous colonisation by new plant species on the fine kimberlite tailings (i.e. 2016–2020) is probably because the fine tailings were more conducive for germination than the coarse kimberlite tailings.

The use of native grass species during restoration improves vegetation cover and the recovery of other native species in alpine environments (Rydgren et al., 2017; Vloon et al., 2022). Consequently, a high diversity of spontaneous emergents, with 15 species in both the fine and the coarse kimberlite tailings, could have resulted from using a tussock grass in the native seed mix to enhance facilitation and effective soil management. *Tenaxia disticha* exhibited nurse effects with its ability to form large tussocks that create germination sites and protect other species against the harsh climate (Nichols et al., 2010; Ntloko et al., 2022). Facilitation successional theory states that planting structurally dominant nurse species can assist colonisation by other native species to restore a disrupted plant community (Nichols et al., 2010). However, the absence of grass species among the 36 emergent plant species that colonised the plots might be partly a result of the fencing of the study site, which restricted the potential biotic dispersal agents of grasses, thereby limiting their recruitment (Klanderud et al., 2017). This is further exacerbated by the reduced abundance of common grasses such as *Festuca caprina* Nees, *Harpochloa falx* (L.f.) Kuntze and *Themeda triandra* Forssk., possibly owing to overgrazing around the mine property (Morris, 2017). Similarly, Ninot et al. (2001) did not find typical species of the mid‐successional stage in the surrounding area of a mine in Spain, probably owing to compromised natural microhabitats. Other important grasses from the reference site at LDM were not recorded (Du Preez et al., 1991). This suggests that the recovery of grassland assemblages within plant communities of the mine tailings might require longer than 5 years in the Drakensberg Afro‐alpine area. We argue that the difference in abundance of plant species in both the fine and the coarse kimberlite tailings is likely a result of environmental filters such as the conditions of the soil, the inter‐species competitive interactions in each growth medium and proximity of mother plants in the natural environment. Furthermore, the observed plant species assemblage could have been a result of geoecological zonation (i.e. the influence of rocky mining material on vegetation patterns), as reported by Craw and Rufaut (2021) on the mine dumps in New Zealand, thus suggesting that other species will only colonise as the environment is ameliorated by these pioneer species over time. Generally, the natural post‐disturbance recovery of vegetation in alpine ecosystems is slow owing to harsh environmental conditions, short growing seasons, and the slow rate of the biological processes (Krautzer et al., 2012; Mokotjomela et al., 2009; Ntloko et al., 2022). Indeed, plant community development is mostly dependent on natural processes in different community successional stages, together with the environmental context that was possibly created during manipulation of the substrates in our study plots of fine and coarse kimberlite tailings (Roman & Cristea, 2012; Vaughn et al., 2010; Vloon et al., 2022).

In conclusion, whereas regular failures have been reported for plant establishment and persistence during rehabilitation, our results represent a first revegetation success for kimberlite dumps in an alpine region using a combination of substrate amelioration and a seed mix of native pioneer plant species. We have shown that the rehabilitation of fine and coarse kimberlite tailings in the alpine area of the Lesotho Drakensberg requires topsoil to ameliorate the kimberlite substrates and, using different combinations of treatments, we also determined the best growth mediums to achieve revegetation. Our results are consistent with previous recommendations to find a suitable combination of different approaches to promote plant establishment. Seeking to comply with the sustainable development model (Nzimande & Chauke, 2012), we have shown that understanding the site conditions and history is critical. Rehabilitation approaches must be scientifically tested to choose the best option to be implemented on‐site. Since the end land use must support biodiversity and domestic livestock farming by local human societies, this study identified plant species and tested different combinations of substrate mixes to determine how sites should be prepared with limited resources to increase the chances of success. Over 5 years of monitoring, we found that a native plant community had established, dominated by forbs, and few grass species which are the main target component of the pasture‐orientated end land use. Nevertheless, these are early days, and this contextual setback can be mitigated by increasing grass species in the seed mix during the large‐scale implementation of rehabilitation strategies at LDM to expedite the achievement of usability of the post‐mining landscapes. Finally, our study confirms that facilitation is important for the development of plant communities and species richness in tailings facilities in an alpine area. Further studies are required to investigate whether native species seed mixes and soil quality across different treatments can be improved so that we can attain a climax grassland community that can deliver the necessary ecosystem services during the end land use.

## AUTHOR CONTRIBUTIONS

**B. R. Ntloko:** Conceptualization (equal); data curation (equal); investigation (equal); methodology (equal); project administration (equal); resources (equal); writing – original draft (equal). **T. M. Mokotjomela:** Conceptualization (equal); data curation (equal); formal analysis (equal); investigation (equal); methodology (equal); supervision (equal); visualization (equal); writing – original draft (equal); writing – review and editing (equal); writing – review and editing (equal). **S. P. Mphafi:** Data curation (equal); formal analysis (equal); investigation (equal); methodology (equal); writing – original draft (equal). **S. J. Siebert:** Conceptualization (equal); funding acquisition (equal); methodology (equal); resources (equal); supervision (equal); visualization (equal); writing – original draft (equal); writing – review and editing (equal).

## Supporting information


Tables S1–S2

## Data Availability

Data: Ntloko et al_Drakensberg mine rehabilitation data submitted with DOI https://doi.org/10.5061/dryad.w3r2280xq. Dryad‐Link: (https://datadryad.org/stash/share/W7LlcNJGaJuoWroYvgJYBIbynZyWigV8guohSfCmb3s).
