# Supplementary material for: Success in restoring native plant communities on kimberlite mining dumps in the Afro‐alpine Drakensberg region of Lesotho
Source: Ecol Evol. 2024 Mar 12;14(3):e11022. doi: 10.1002/ece3.11022 (PMC10932747; doi:10.1002/ece3.11022)
Supplement: Supplementary file 1 — Tables S1–S2 [file ECE3-14-e11022-s001.doc]

**Supporting material:**

Table S1: Wald statistics for comparison of plant abundance (i.e., counts of individual plants in each growth medium) in different treatments composed of fine kimberlite tailings (FT) and coarse kimberlite tailings (CT) during the 2016 to 2020 period of monitoring. ‘Soil’ refers to topsoil from stockpile; WR = waste rock. Superscript “a” represents a parameter that was selected as a reference for comparison of the significance.

|  | **Parameter** | **B** | **Std error** | **Hypothesis test** | | |
| --- | --- | --- | --- | --- | --- | --- |
|  |  |  |  | **Wald chi-square** | **Df** | ***Significance*** |
|  | **Intercept** | **4.067** | **0.1553** | **685.684** | **1** | **P < 0.0001** |
| **Treatments (fine kimberlite tailings)** | FT | -0.754 | 0.1114 | 45.788 | 1 | P < 0.0001 |
|  | FT+CT(100mm)+soil (100mm) | -0.380 | 0.1092 | 12.118 | 1 | P < 0.0001 |
|  | FT+CT(250mm) | -0.783 | 0.1131 | 47.931 | 1 | P < 0.0001 |
|  | FT+CT(250mm)+soil (100mm) | 0^a^ | . | . | . | . |
| **Years (fine kimberlite tailings)** | 2016 | -1.040 | 0.1617 | 41.423 | 1 | P < 0.0001 |
|  | 2017 | -0.857 | 0.1448 | 35.043 | 1 | P < 0.0001 |
|  | 2018 | -0.379 | 0.1445 | 6.868 | 1 | P < 0.001 |
|  | 2019 | -1.294 | 0.1469 | 77.655 | 1 | P < 0.0001 |
|  | 2020 | 0^a^ | . | . | . |  |
|  | **Intercept** | **2.949** | **0.1515** | **378.993** | **1** | **P < 0.0001** |
| **Treatments (coarse kimberlite tailings)** | CT | -1.599 | 0.1290 | 153.765 | 1 | P < 0.0001 |
|  | CT+soil(100mm) | -.391 | 0.1082 | 13.025 | 1 | P < 0.0001 |
|  | CT+WR(200mm) | -1.183 | 0.1189 | 98.975 | 1 | P < 0.0001 |
|  | CT+WR(200mm)+soil(100mm) | 0^a^ | . | . | . | . |
|  |  |  |  |  |  |  |
| **Years (coarse kimberlite tailings)** | 2016 | -1.491 | 0.1699 | 77.032 | 1 | P < 0.0001 |
|  | 2017 | -1.823 | 0.1481 | 151.519 | 1 | P < 0.0001 |
|  | 2018 | -1.697 | 0.1476 | 132.240 | 1 | P < 0.0001 |
|  | 2019 | -1.646 | 0.1269 | 168.126 | 1 | P < 0.0001 |
|  | 2020 | 0^a^ | . | . | . | . |
|  |  |  |  |  |  |  |

Table S2: Plant species diversity indices for fine and coarse kimberlite tailings at Letšeng Diamond Mine, measured using Shannon-Weiner and Simpson (1949) procedures.

| **Site** | **Shannon-Weiner indices** | | | **Simpson scores** | |
| --- | --- | --- | --- | --- | --- |
|  | ***H*** | ***H-max*** | ***Equitability*** | D |  |
| Fine kimberlite tailings | 3.1 | 3.4 | 0.91 | 0.95 |  |
|  |  |  |  |  |  |
| Coarse kimberlite tailings | 3.2 | 3.5 | 0.90 | 0.95 |  |
|  |  |  |  |  |  |
